# Formulation and Evaluation of Antibacterial Creams and Gels Containing Metal Ions for Topical Application

**DOI:** 10.1155/2016/5754349

**Published:** 2016-11-03

**Authors:** Mei X. Chen, Kenneth S. Alexander, Gabriella Baki

**Affiliations:** Department of Pharmacy Practice, College of Pharmacy and Pharmaceutical Sciences, University of Toledo, 3000 Arlington Ave., Toledo, OH 43614, USA

## Abstract

*Background.* Skin infections occur commonly and often present therapeutic challenges to practitioners due to the growing concerns regarding multidrug-resistant bacterial, viral, and fungal strains. The antimicrobial properties of zinc sulfate and copper sulfate are well known and have been investigated for many years. However, the synergistic activity between these two metal ions as antimicrobial ingredients has not been evaluated in topical formulations.* Objective.* The aims of the present study were to (1) formulate topical creams and gels containing zinc and copper alone or in combination and (2) evaluate the* in vitro* antibacterial activity of these metal ions in the formulations.* Method.* Formulation of the gels and creams was followed by evaluating their organoleptic characteristics, physicochemical properties, and* in vitro* antibacterial activity against* Escherichia coli* and* Staphylococcus aureus*.* Results.* Zinc sulfate and copper sulfate had a strong synergistic antibacterial activity in the creams and gels. The minimum effective concentration was found to be 3 w/w% for both active ingredients against the two tested microorganisms.* Conclusions.* This study evaluated and confirmed the synergistic* in vitro* antibacterial effect of copper sulfate and zinc sulfate in a cream and two gels.

## 1. Introduction

Topical skin infections commonly occur and often present therapeutic challenges to practitioners, despite the numerous existing antimicrobial agents available today. The necessity for developing new antimicrobial means has increased significantly due to growing concerns regarding multidrug-resistant bacterial, viral, and fungal strains [1–4]. Consequently, attention has been devoted to safe, new, and/or alternative antimicrobial materials in the field of antimicrobial chemotherapy.

Common examples for topical skin infections include diaper rash, cold sores, and tinea (also called pityriasis) versicolor. Diaper rash is a form of irritant contact dermatitis. It is one of the most common dermatological conditions encountered in babies while using diapers [5] and is estimated to occur in 7–35% of babies between the ages of 9 and 12 months [6]. Its development is multifactorial, including skin wetness, friction, skin irritants, and pH change, which favors the growth of microorganisms including* Candida*,* Staphylococcus*, and* Streptococcus *[7]. It has been shown that zinc and copper ions have antimicrobial activity against* Staphylococcus aureus* and* Candida albicans* [8]. Cold sores (also known as herpes labialis) are a common viral infection occurring on the lips, primarily caused by herpes simplex virus (HSV) type 1 [9]. Studies have shown that zinc and copper salts exhibit inactivation of HSV both* in vivo* and* in vitro *[10–13]. Zinc sulfate was found to have an antimicrobial effect in treating cold sores [14]. The molecular mechanism of its therapeutic effect was found to be the drastic inactivation of free virus in skin tissues, intercellular vesicles, and blisters [15]. Pityriasis versicolor is a superficial fungal infection of the skin, usually caused by* Malassezia* species. It is one of the most common skin diseases in tropical and subtropical areas and is characterized by fine scaly patches and macules [16]. Both zinc sulfate and copper sulfate have been found to be effective in treating this disease [17, 18].

In recent years, a number of metal ions have been studied as potential antimicrobial agents, including silver [19], copper [20], zinc [21], iron [22], magnesium [23], and titanium [24]. Zinc, alone or as an adjuvant, has been found to be advantageous in a number of dermatological infections and inflammatory diseases owing to its modulating actions on macrophage and neutrophil functions, natural killer cell/phagocytic activity, and various inflammatory cytokines. Zinc sulfate has been studied* in vivo* in a number of diseases, including warts [25], herpes genitalis [26], pityriasis versicolor [18], and acne vulgaris [27] in varying concentrations. Copper is well known for its antimicrobial properties. It has been used as an algicide, germicide, and fungicide for decades. Several antimicrobial mechanisms of copper were proposed in recent articles, including reactive hydroxyl radical formation leading to damaged cell integrity, denaturation of DNA by binding of copper to protein molecules, and inactivation of enzymes and obstruction of functional groups of proteins from displacement of essential ions [28–30]. Additionally, topically applied copper sulfate and hypericum perforatum were found to be efficacious* in vivo* in the treatment of herpes skin lesions [13].

The antimicrobial activity of zinc sulfate and copper sulfate has been investigated for many years. However, the synergistic activity between these two metal ions as antimicrobial ingredients has not been evaluated in topical formulations. The aim of the present study was to formulate topical creams and gels containing zinc sulfate or copper sulfate, and a combination of these, and to evaluate the* in vitro* antibacterial activity of these metal salts in the formulations against* Escherichia coli* and* Staphylococcus aureus*. The* in vitro* antibacterial activity of the formulated products was also compared to commercial products available for the treatment of diaper rash and cold sores.

Incorporating metal ions such as zinc and copper often creates a formulation challenge due to the high reactivity of these ions. Even trace amounts of metal ions are able to catalyze oxidation reactions in fatty compounds in products, leading to deterioration including odor formation, color change, and physical and/or chemical instability [31]. Metal ion reactions with the ingredients in the formulations can affect the quality, efficacy, consumer appeal, and shelf-life of formulations. Stability of product and of the antibacterial activity was studied for 12 weeks at two different temperatures in two different containers.

## 2. Materials and Methods

### 2.1. Materials

Copper sulfate pentahydrate was purchased from Fagron, Inc. (St. Paul, MN). Zinc sulfate heptahydrate, Carbomer 940, refined corn oil, almond oil sweet, lecithin soya granular, glycerin, and propylene glycol were purchased from Letco Medical (Decatur, AL). (ι)-Carrageenan was purchased from Sigma-Aldrich (St. Louis, MO). Hypromellose (Benecel, K4M PHARM, also known as hydroxypropylmethyl cellulose, HPMC), and Prolipid 141 (a mixture of glyceryl stearate, behenyl alcohol, palmitic acid, stearic acid, lecithin, lauryl alcohol, myristyl alcohol, and cetyl alcohol) were received as gifts from Ashland (Wilmington, DE). Kollidon® 90F (poly vinylpyrrolidone, PVP) was obtained from BASF (Ludwigshafen, Germany). Poloxamer 407 was purchased from PCCA (Houston, TX). FlexiThix™ (2-pyrrolidinone-1-ethenyl homopolymer) was received as a free sample from ISP Technologies, Inc. (Wayne, NJ). Xanthan gum, guar gum, methylparaben, propylparaben, butylated hydroxytoluene (BHT), and citric acid monohydrate were obtained from Spectrum Chemical (Gardena, CA). Medium chain triglycerides (MCT) were obtained from Mead Johnson & Company (Evansville, Indiana). Soybean oil, Cithrol™ GMS 40 (glyceryl stearate), Arlacel™ 165 (a mixture of glyceryl stearate and PEG-100 stearate), Tween 60, and Span 80 were received as free samples from Croda, Inc. (Edison, NJ). PEG-16 Macadamia and PEG-10 Sunflower were obtained from FloraTech (Gilbert, Arizona). Cocoa butter was a gift from Koster Keunen, Inc. (Watertown, CT). Cetyl alcohol, stearic acid, stearyl alcohol, and isopropyl myristate were obtained from Sherman Research Labs (Toledo, OH). Coconut oil was purchased from Spectrum Organic Products, (Melville, NY). Tefose HC (a mixture of cetyl alcohol, glyceryl stearate, ceteth-20, and steareth-20) was a free sample from Gattefossé (Saint-Priest Cedex, France). PEG-8 beeswax was a gift from Koster Keunen, Inc. (Watertown, CT). Urea was purchased from Gallipot®, Inc. (St. Paul, MN). Triethanolamine was purchased from Making Cosmetics (Snoqualmie, WA). Mueller-Hinton agar and gentamicin 10 *μ*g standard discs were purchased from Becton, Dickinson and Company (Sparks, MD). The marketed products included Equate® Diaper Rash Relief Cream (distributed by Walmart, Inc.), Nexcare™ Cold Sore Treatment Cream (distributed by 3M), and Campho-Phenique® Cold Sore Treatment Gel (distributed by Bayer Health Care LLC), which were all purchased at a local Walmart store (Toledo, OH). All ingredients used in the various formulations can be found in Tables 1 and 2.

### 2.2. Methods

#### 2.2.1. Formulation of the Topical Cream

The oil phase was prepared by melting the waxes at 75°C and mixing the ingredients uniformly. The aqueous phase was prepared by dissolving the water-soluble ingredients in deionized water. The water phase was warmed to 75–80°C until all ingredients were dissolved. When the water and oil phase were at the same temperature, the aqueous phase was slowly added to the oil phase with moderate agitation and was kept stirred until the temperature dropped to 40°C. The emulsion was cooled to room temperature to form a semisolid cream base. Zinc sulfate and copper sulfate were dissolved in warmed deionized water, and the solutions were added to the cream base using an overhead stirrer (Talboys Engineering Corp, Emerson, NJ). The mixture was stirred for 15 min until the formulation became uniform. The drug-loaded cream was preserved with paraben concentrate. The exact concentration of each ingredient is shown in Table 1.

#### 2.2.2. Formulation of the Topical Gels

When using (ι)-carrageenan, xanthan gum, and guar gum, the powder polymers were dispersed in 75°C warm deionized water with stirring. When all the polymers were dissolved, the mixture was removed from the hot plate. The desired amount of zinc sulfate and copper sulfate was dissolved in the clear gel with intensive stirring. The mixture was then cooled to room temperature and preserved with paraben concentrate.

In formulations where HPMC was the thickening agent, the polymer was dispersed in 75°C warm deionized water with stirring. The resulting solution was stored at room temperature overnight until a clear gel formed. Zinc sulfate crystals and then copper sulfate crystals, after complete dissolution, were dispersed into the gel with intensive agitation. Preservative was added to the formulation in the last step.

Poloxamer was dissolved in cold water and stored under refrigerated conditions at 4°C for a night. The oil phase was prepared by mixing lecithin and isopropyl myristate in a 1 : 1 ratio. The mixture was stored at room temperature overnight for the complete dissolution of lecithin. The active ingredients were then added directly to the aqueous phase. The gel was prepared by mixing 1 part of oil phase with 4 parts of aqueous phase (poloxamer gel) using a vortex mixer (VORTEX-T, Genie® 2, Bohemia, NY).

Kollidon 90F, FlexiThix, and Carbomer 940 were directly dispersed into deionized water at room temperature with intensive agitation. Active ingredients were incorporated into the gel uniformly. In order for Carbomer 940 to form a gel, triethanolamine was added to neutralize the pH to 6–6.5.  Table 2 shows the amount of ingredients used for the gels.

#### 2.2.3. Physical Evaluation of the Topical Formulations

*(1) Organoleptic Characteristics.* All blank formulations (i.e., formulations without any active ingredients or preservatives) and drug-loaded formulations were tested for physical appearance, color, texture, phase separation, and homogeneity. These characteristics were evaluated by visual observation. Homogeneity and texture were tested by pressing a small quantity of the formulated cream and gels between the thumb and index finger. The consistency of the formulations and presence of coarse particles were used to evaluate the texture and homogeneity of the formulations. Immediate skin feel (including stiffness, grittiness, and greasiness) was also evaluated.

*(2) Spreadability.* Spreadability of the formulations was determined by measuring the spreading diameter of 1 g of sample between two horizontal glass plates (10 cm × 20 cm) after one minute. The standard weight applied to the upper plate was 25 g. Each formulation was tested three times.

*(3) pH Values. *One gram of each formulation (including the blank, i.e., formulation without any active ingredients or preservatives, and drug-loaded formulation) was dispersed in 25 mL of deionized water, and the pH was determined using a pH meter (Mettler-Toledo Ingold Inc., Billerica, MA). Measurements were made in triplicate. The pH meter was calibrated with standard buffer solutions (pH 4, 7, and 10) before each use.

*(4) Viscosity Measurement.* A Brookfield viscometer DV-I (Brookfield Engineering Laboratories, Middleboro, MA) was used with a concentric cylinder spindle #29 to determine the viscosity of the different topical formulations. The tests were carried out at 21°C. The spindle was rotated at 0, 0.5, 1, 2, 2.5, 4, 5, 10, 20, 50, and 100 rpm values. All measurements were made in triplicate.

#### 2.2.4. *In Vitro* Antibacterial Activity

*(1) Preparation of Mueller-Hinton (MH) Agar Plates.* Mueller-Hinton (MH) agar medium was prepared according to the manufacturer's instructions and autoclaved for 20 minutes at 20 psi. After autoclaving, the agar medium was cooled to 40–45°C in a water bath. Sixty mL of the cooled agar medium was poured onto the prepared 150 × 15 mm petri dish. The agar was allowed to cool to room temperature and stored in a refrigerator (2–8°C) until used.

*(2) Preparation of Inoculum*.* Escherichia coli* (ATCC 25922) and* Staphylococcus aureus* (ATCC 29213) were used to evaluate the antibacterial activity of the topical formulations containing zinc sulfate and copper sulfate. The microorganisms were subcultured the previous day to ensure that the tested microorganisms were in their log phase of growth and to ensure the validity of the results. One or two isolated colonies of the tested microorganisms were touched using a sterile cotton swab. The microorganisms were suspended in 2 mL of sterile saline medium and vortexed well until a uniform suspension was obtained. The turbidity of the suspension was measured at 625 nm using a UV-Vis spectrophotometer (Thermo Scientific, Waltham, MA). The turbidity of the suspension was adjusted to a 0.5 McFarland standard by adding more microorganism if the suspension was too light or diluting with sterile saline if the suspension was too heavy. The suspension was prepared before inoculating the microorganisms on the agar plate.

*(3) Inoculation of the MH Plate.* To inoculate the MH agar plates, a sterile cotton swab was dipped into the suspension and streaked over the surface of the agar plates. This procedure was repeated three times; each time, the plate was rotated approximately 60 degrees to ensure even distribution of the inoculum [32]. The plates were then allowed to dry at room temperature for 5 min before applying the drug.

*(4) Preparation of Agar Well Diffusion Assay.* The dried inoculated MH agar plates prepared above were used to perform the agar well diffusion assay. A sterile cork borer was used to make the wells by punching holes on the inoculated MH agar plates. Each well was 5 mm in diameter, and the cut-out of the agar was removed using a sterile needle. A desired amount of the formulations was weighed and placed into each well on an analytical balance. Gentamicin 10 *μ*g standard discs were used as a control to ensure that the agar medium was appropriate to support the growth of the microorganism beyond the zone of inhibition. The gentamicin standard disc was placed and pressed gently onto the same inoculated agar plate by using sterile forceps. The inoculated agar plate was incubated at 37°C for 18 hours. The observed diameters of the zones of inhibition were measured by using a ruler to the nearest millimeter.

First, in order to observe how effective the active ingredients were alone versus combined, 20 *μ*L of 3% copper sulfate, 3% zinc sulfate, 6% copper sulfate, 6% zinc sulfate, and 3 + 3% copper sulfate and zinc sulfate combined solutions were tested for antibacterial activity against* E. coli* and* S. aureus*. Next, the selected cream and gel formulations containing both active ingredients in a series of concentrations (including 0, 0.1, 0.25, 0.5, 1, 2, and 3% of each ingredient) were tested for antibacterial activity against* E. coli* and* S. aureus* to evaluate their effective concentration. Gentamicin 10 *μ*g standard disc was used as the control. The sample size directly measured into the wells was 80.2 ± 0.3 *μ*g in this study. Finally, the selected cream and gel formulations were directly compared to the marketed products, including Nexcare Cold Sore Treatment Cream, Campho-Phenique Cold Sore Treatment Gel, and Equate Diaper Rash Relief Cream, in terms of their antibacterial activity against* E. coli* and* S. aureus*. The sample size directly measured into the wells was 72.3 ± 1 *μ*g in this study.

#### 2.2.5. Stability Study

The antibacterial activity of all selected drug-loaded formulations was tested against* E. coli* using the above-described agar well diffusion assay for 12 weeks (measurements were made on day 1, week 3, week 6, week 9, and week 12). The antibacterial activity of the formulations was compared for samples stored at room temperature (25°C) and in the refrigerator (4°C) as well as those packaged into glass containers versus plastic containers. In addition to the antibacterial activity, pH values, color, physical appearance, and texture were also tested during the 12 weeks with the above-described methods.

#### 2.2.6. Statistical Analysis

Statistical analysis of data was performed using one-way ANOVA (Tukey's* post hoc* test). A difference was considered statistically significant when *p* < 0.05.

## 3. Results

From the twenty different creams formulated, C1 was selected as the final formulation for further testing. Eighteen different gels were formulated in this study, from which G1 and G5 were selected as optimal formulations for further evaluation.

### 3.1. Physical Evaluation of Topical Cream and Gels

#### 3.1.1. Organoleptic Characteristics

The organoleptic properties, including physical appearance, color, texture, phase separation, homogeneity, and immediate skin feel of the selected topical formulations, are displayed in Table 3. Results showed that the cream and both gels had a cosmetically appealing appearance and smooth texture, and they were all homogenous with no signs of phase separation. All formulations were blue due to copper sulfate.

#### 3.1.2. Spreadability

**Figure 1:**
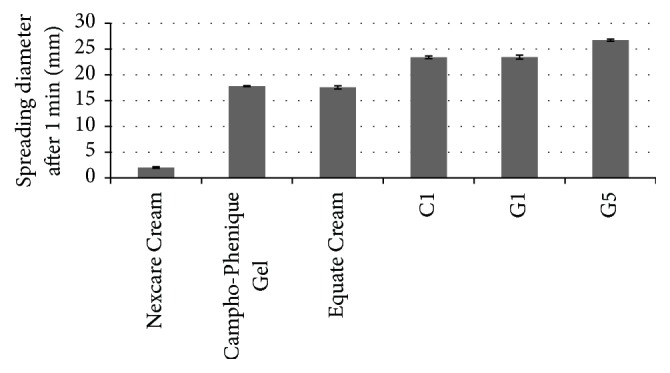
Spreadability values for the selected cream (C1) and gels (G1 and G5) compared to various marketed products (*n* = 3, results shown as mean ± SD).

Spreadability of semisolid formulations, that is, the ability of a cream or gel to evenly spread on the skin, plays an important role in the administration of a standard dose of a medicated formulation to the skin and the efficacy of a topical therapy.  Figure 1 shows the spreading values, that is, diameters observed for the formulations, after one minute. The values refer to the extent to which the formulations readily spread on the application surface by applying a small amount of shear. Results indicated that our cream and gels had comparable spreadability to that of commercial products used as comparators in the study.

#### 3.1.3. pH Values

The pH values for the blank and drug-loaded cream and gels are shown in Table 4. The pH of the formulations decreased when the active ingredients were added to the bases. The pH of the skin normally ranges from 4 to 6. The pH of the cream was more acidic than that of the skin, while the gels' pH values were similar to the skin's normal pH value. The pH values of the formulations did not change significantly over the period of 12 weeks.

#### 3.1.4. Viscosity Measurement

**Figure 2:**
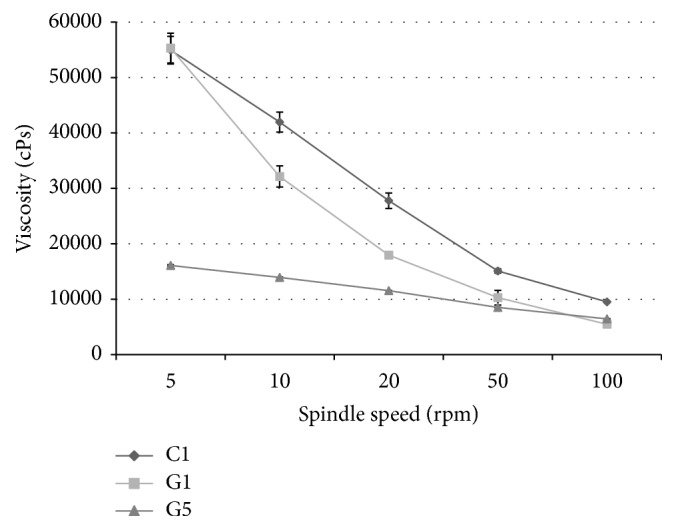
Viscosity curves for the selected cream (C1) and gels (G1 and G5) (*n* = 3, results shown as mean ± SD).

Viscosity values for the drug-loaded cream and gels are shown in Figure 2. All products had a pseudoplastic behavior, as expected. C1 and G1 had a similar viscosity curve, while G5 had a lower initial viscosity.

#### 3.1.5. *In Vitro* Antibacterial Activity

The* in vitro* antibacterial study was performed by measuring and comparing the diameter of zones of inhibition (in mm) for the various products. The zone of inhibition can be defined as the clear region around the well that contains an antimicrobial agent. It is known that the larger the zone of inhibition, the more potent the antimicrobial agent.

**Figure 3:**
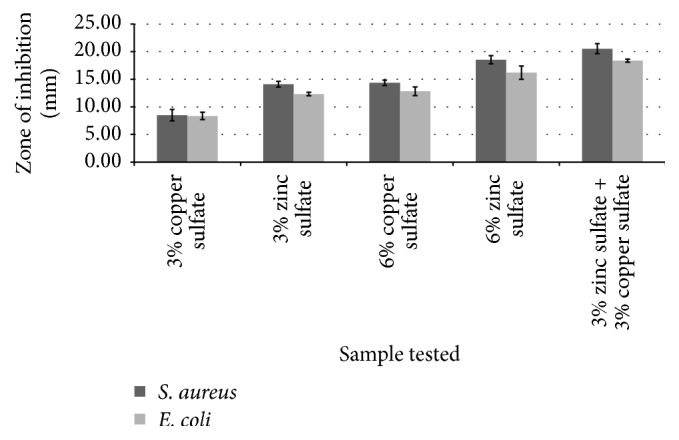
Antimicrobial activity of copper sulfate and zinc sulfate solutions in various concentrations against* Escherichia coli* and* Staphylococcus aureus* (*n* = 3, results shown as mean ± SD) (*p* = 0.05).

In the first step, the two active ingredients' antibacterial activity was measured individually and combined against* E. coli* and* S. aureus*. It can be concluded from the results that the antibacterial activity of zinc sulfate was higher than that of copper sulfate against the tested microorganisms (Figure 3). Results also confirmed that copper sulfate and zinc sulfate have a synergistic activity, as shown by their larger zone of inhibition against tested microorganisms (*p* = 0.05).

**Figure 4:**
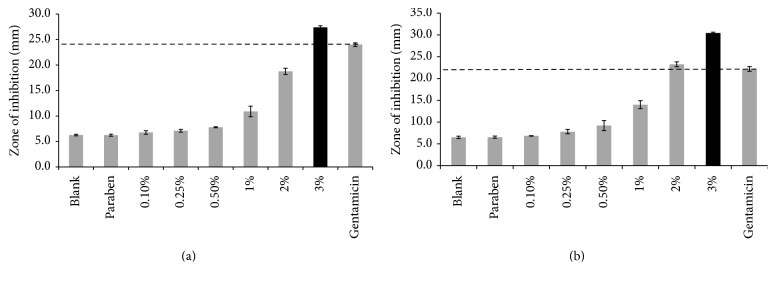
Antimicrobial activity of the selected cream (C1) containing copper sulfate and zinc sulfate in various concentrations against (a)* Escherichia coli* and (b)* Staphylococcus aureus* (*n* = 3, results are shown as mean ± SD) (*p* = 0.05).

In the next step, the antibacterial activity of the selected cream (C1) and gels (G1 and G5) with varying amounts of active ingredients (0, 0.1, 0.25, 0.5, 1, 2, and 3%) was studied against* E. coli* and* S. aureus*. The results are shown in Table 5 and Figure 4. The blank formulation did not contain any active ingredients or any preservative. The formulation named paraben contained preservative but no active ingredients. Gentamicin 10 *μ*g standard disc was used as the control in the study. No zone of inhibition was observed for the blank, paraben, and 0.1% strength formulations for either the cream or the gels. The zones of inhibition increased as the concentration of copper sulfate and zinc sulfate increased. This indicated that the antibacterial activity of copper sulfate and zinc sulfate increased against* E. coli* and* S. aureus* as the concentration of the actives was increased. As for the C1 formulation, the antibacterial activity of the sample containing 2% active ingredients was as good as that of the control (gentamicin) against* S. aureus*, while the antibacterial activity of the sample containing 3% active ingredients was statistically significantly higher than the control and the rest of the samples against both* E. coli* and* S. aureus *(*p* = 0.05). A visual representation of these results for C1 can be found in Figure 4. In the case of G1, similar results were seen. Samples containing 2% active ingredients had a similar antibacterial activity to that of the control, while the samples containing 3% active ingredients had statistically significantly higher activity against both microorganisms. As for G5, both the 2% and 3% samples had a similar antibacterial activity as the control against* E. coli*, while only the samples containing 3% of each active ingredient had an antibacterial activity comparable to the control against* S. aureus*. Based on the results, it can be concluded that both active ingredients have to be present in a concentration of at least 3% to achieve a similar or better antibacterial activity as the control against* E. coli* and* S. aureus*.

**Figure 5:**
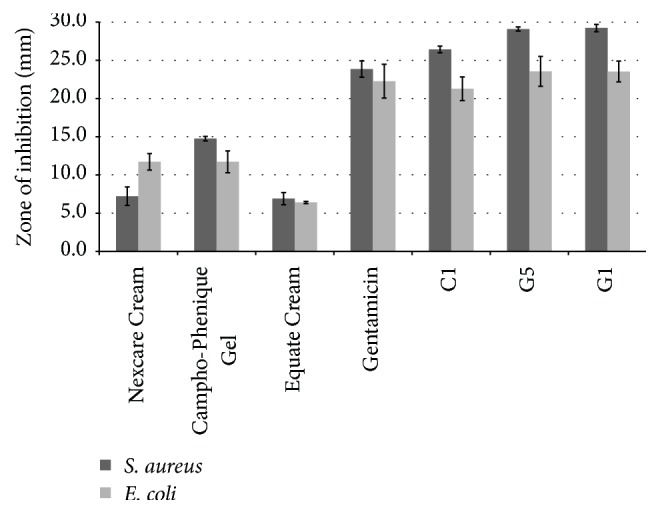
Antimicrobial activity of the selected cream (C1), gels (G1 and G5), and three marketed products (Nexcare Cold Sore Treatment Cream, Campho-Phenique Cold Sore Treatment Gel, and Equate Diaper Rash Relief Cream) against* Escherichia coli* and* Staphylococcus aureus* (*n* = 3, results shown as mean ± SD).

The final* in vitro* antimicrobial study was performed to compare the antibacterial activity of the selected formulations to those of marketed products against* E. coli* and* S. aureus*. As there is currently no marketed product available with zinc sulfate and copper sulfate, two commercially available cold sore gels and a diaper rash cream, Nexcare Cold Sore Treatment Cream, Campho-Phenique Cold Sore Treatment Gel, and Equate Diaper Rash Relief Cream, were used as comparators in the study. Nexcare Cold Sore Treatment Cream contains the following active ingredients: benzocaine as an external analgesic and allantoin as a skin protectant. The active ingredients in Campho-Phenique Cold Sore Treatment Gel are camphor and phenol as pain relievers or antiseptics, and, in Equate Diaper Rash Relief Cream, zinc oxide is a skin protectant. The results of this study are displayed in Figure 5. Results indicated that the antibacterial activity of C1, G1, and G5 was similar to that of the control (gentamicin), while the marketed products had a significantly lower activity against the two tested microorganisms. G1 and G5 had higher antibacterial activity against the tested bacteria than C1, which may be related to the composition of these products. The cream formulation contained oily components, which are immiscible with water and may slow down the diffusion of the drugs from the cream base.

### 3.2. Stability Study

**Figure 6:**
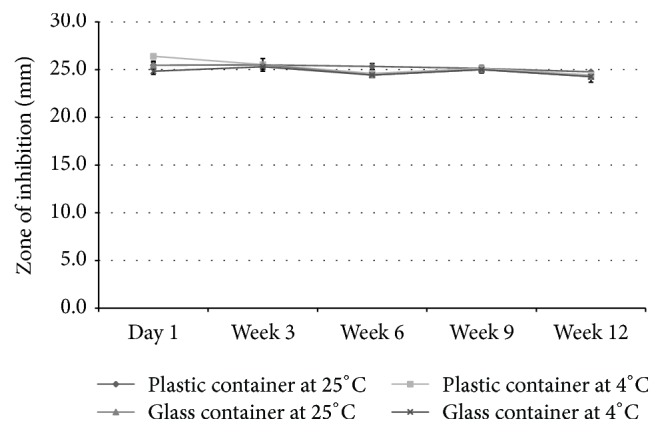
Antimicrobial activity of one of the selected gels (G5) during the stability testing compared to the control (gentamicin) against* Escherichia coli* (*n* = 3, results shown as mean ± SD).

All formulations maintained their blue color and intensity of color for 12 weeks in all storage conditions. Similarly, the physical appearance, homogeneity, and texture of all formulations remained the same by the end of the storage period. None of the formulations showed signs of physical or chemical instability in any of the containers or at any of the temperatures. The antibacterial activity of all formulations was maintained for 12 weeks in both containers and at both temperatures. Results are shown in Figure 6 for G5 as well as in Table 6 for C1 and G1.

## 4. Discussion

Twenty different cream bases (C1–C20) were formulated using different ingredients in varying concentrations. After incorporating the active ingredients into the bases, the physical stability of a number of bases was affected negatively by the metal ions, leading to creaming and breaking of the emulsions. Formulations C5–C20 suffered from such issues; therefore, they were discontinued from further characterization. Formulations C1–C4 were formulated with the same ingredients, but with varied concentrations of the emulsifiers and thickeners. These four creams had similar consistency, and no apparent change in their physical appearance was observed after adding the metal salts. Based on the overall evaluation for physical appearance and immediate skin feel, C1 was selected as the final formulation for further testing.

In addition to the creams, eighteen different gels (G1–G18) were formulated using eight different gelling agents. Only two polymers, namely, carrageenan and HPMC, proved to be optimal gelling agents for the metallic active ingredients used. G1 and G2 were formulated using carrageenan. These gels incorporated the active ingredients well. They had similar texture, consistency, and viscosity. The appearance, viscosity, and skin feel provided by G1 was considered better for a topical gel; therefore, it was selected for further testing. G3–G6 were formulated using different concentrations of HPMC. Out of these four samples, only G5 provided an optimal gel. To be considered optimal in this study, a gel (1) had to be homogenous without showing signs of physical or chemical instability; (2) had to be able to dissolve and keep the active ingredients in a dissolved form without precipitating or aggregating; and (3) had to have a high enough viscosity not to flow off the skin during/after application. In the case of G3 and G4, precipitation or aggregation of the polymers was observed when the active ingredients were added to the 5% HPMC gel. The explanation for this is that the amount of water used in these gels was not enough to dissolve the crystalline actives and keep the gelling agent dispersed. G4 contained more water compared to G3; however, even that higher amount did not prove to be enough for a stable formulation. G5 met all our requirements; therefore this formulation was selected for further testing. G6 had a too low viscosity, which was not deemed appropriate for a topical gel. As for the other polymers used, including xanthan gum, guar gum, poloxamer 407, Kollidon 90F, FlexiThix, and Carbomer 940, the gels' physical stability was compromised when the active ingredients were added to the gel bases.

The three selected formulations were optimal in terms of their appearance, homogeneity, and viscosity. Previous studies [6–9] indicated that both active ingredients had antibacterial activity, which this study confirmed. The two metal salts had a synergistic activity when combined in the creams and gels, which was also confirmed by our study. The second antibacterial study indicated that both zinc sulfate and copper sulfate have to be present in at least a 3% concentration in order to achieve similar or better results against the tested microorganisms than gentamicin, which was used as the control. As there are no marketed products available today with copper sulfate and/or zinc sulfate, commercial products for the treatment/prevention of cold sores and diaper rash with active ingredients other than copper sulfate and zinc sulfate were used as comparators in the final antibacterial study. This limited study showed that the formulated creams and gels had a significantly higher antibacterial activity against the two tested microorganisms than the marketed products. With a properly planned and conducted follow-up study using additional microorganisms—preferably fungi, bacteria, and viruses—the true antimicrobial activity of the products could be evaluated, and a more realistic comparison could be made.

The stability study indicated that both gels and the cream were able to maintain their integrity for 12 weeks without showing signs of instability. Additionally, the main function, that is, antibacterial activity of all formulations, remained stable for the tested time period, which is promising. As discussed in the introduction, incorporating metal salts into emulsions and gels can be a challenging task for formulators due to the high reactivity of these salts. Many of our formulations were affected by the metal ions, which resulted in precipitation, phase separation, color change, and other forms of instability. Only a few formulations, that is, the selected creams and gels, were able to remain stable after incorporating the active ingredients into them.

## 5. Conclusions

In this study, various creams and gels were formulated with copper sulfate and zinc sulfate, which act as antimicrobial agents. During the formulation process, the quality, appearance, and stability of many creams and gels were affected by the highly reactive metal ions. A cream and two gels were found to be optimal for our purpose, and these were selected for further evaluation based on their physical properties,* in vitro* antibacterial activity, and product stability. Although only a small fraction of the formulated products were deemed optimal, a great achievement is that the integrity, pH values, texture, appearance, and antibacterial activity of these selected products were maintained for 12 weeks.

A major finding of this study is that copper sulfate and zinc sulfate have a synergistic antibacterial activity in creams and gels. The minimum effective concentration* in vitro* was found to be 3% for both active ingredients. A properly planned and conducted* in vitro* follow-up study could confirm the antimicrobial activity of the formulations against other microorganisms, and a more realistic comparison could be made between our products and marketed products for various skin conditions.

## Figures and Tables

**Table 1:** Composition of the topical cream formulations.

	Amount of each ingredient (%); formulations are coded from 1 to 20																			
Ingredients	C1	C2	C3	C4	C5	C6	C7	C8	C9	C10	C11	C12	C13	C14	C15	C16	C17	C18	C19	C20
Copper sulfate	3																			
Zinc sulfate	3																			
Corn oil	4	4	4	4	—	—	—	—	—	—	—	—	—	—	—	—	—	—	—	—
MCT	4	4	4	4	—	3	3	3	5	5	4	4	—	—	—	6	—	4	4	4
Sweet Almond oil	4	4	4	4	5	4	4	5	—	—	—	—	10	10	6	—	5	4	4	4
Coconut oil	—	—	—	—	—	—	—	—	5	5	4	4	—	—	—	4	5	4	4	4
Cocoa butter	—	—	—	—	—	—	—	—	—	—	—	—	—	—	—	—	3	—	—	—
Soy bean oil	—	—	—	—	5	6	6	6	5	5	4	4	—	—	6	4	4	—	—	—
PEG-16 Macadamia	—	—	—	—	—	—	—	—	—	—	—	—	—	—	—	4	—	—	—	—
PEG-10 Sunflower	—	—	—	—	—	—	—	—	—	—	—	—	2	2	—	—	—	—	—	—
Tefose HC	4	5	6	6	—	—	—	—	—	—	—	—	—	—	—	—	—	—	—	—
Prolipid 141	5	6	5	7	—	—	—	—	—	—	—	—	—	—	—	—	—	—	—	—
PEG-8 Beeswax	7	8	6	6	—	—	—	—	—	4	6	7	—	—	—	—	—	7	6	7
Cithrol GMS 40	5	6	6	6	—	—	—	—	—	—	—	—	—	—	—	—	—	—	—	3
Span 80	—	—	—	—	—	—	—	—	—	—	—	—	1.05	2.25	1.57	1.42	1.77	—	—	—
Tween 60	—	—	—	—	—	—	—	—	—	—	—	—	0.95	2.75	2.43	2.58	2.23	—	—	—
Stearyl Alcohol	—	—	—	—	—	—	—	—	—	—	—	—	—	—	—	4	5	—	5	2
Stearic acid	—	—	—	—	3	2	3	4	4	4	4	5	2	2	5	—	—	4	—	—
Cetyl alcohol	—	—	—	—	5	4	5	7	6	6	6	7	2	4	5	3	5	6	6	6
Arlacel 165	—	—	—	—	5	5	5	5	5	5	5	5	—	—	—	—	—	5	5	5
Urea	—	—	—	—	3	—	—	—	—	—	—	—	4	4	3	3	—	—	—	—
2% HPMC gel	—	—	—	—	—	—	—	—	—	—	—	—	—	—	—	—	—	5	—	—
Xanthan gum	—	—	—	—	—	0.5	0.25	0.25	0.25	0.25	0.25	0.25	—	—	0.5	0.5	—	—	—	—
Carrageenan	0.35	0.35	0.35	0.35	—	—	—	—	—	—	—	—	—	—	—	—	—	—	—	—
Glycerin	5																			
Citric acid	1																			
BHT	0.05																			
DI water	qs ad to 100																			

MCT: medium chain triglycerides; HPMC: hypromellose; BHT: butylated hydroxytoluene.

**Table 2:** Composition of the topical gel formulations.

	Amount of each ingredient (%); formulations are coded from 1 to 18																	
Ingredients	G1	G2	G3	G4	G5	G6	G7	G8	G9	G10	G11	G12	G13	G14	G15	G16	G17	G18
Zinc sulfate	3																	
Copper sulfate	3																	
ι-Carrageenan	2	1	—	—	—	—	—	—	—	—	—	—	—	—	—	—	—	—
5% HPMC gel	—	—	qs 100	qs 100	50	25	—	—	—	—	—	—	—	—	—	—	—	—
Xanthan gum	—	—	—	—	—	—	2	—	—	—	—	—	—	—	—	—	—	—
Guar gum	—	—	—	—	—	—	—	2	—	—	—	—	—	—	—	—	—	—
Poloxamer 407	—	—	—	—	—	—	—	—	32	24	16	—	—	—	—	—	—	—
Lecithin	—	—	—	—	—	—	—	—	10	10	10	—	—	—	—	—	—	—
Isopropyl myristate	—	—	—	—	—	—	—	—	10	10	10	—	—	—	—	—	—	—
Kollidon 90F	—	—	—	—	—	—	—	—	—	—	—	30	20	10	—	—	—	—
FlexiThix	—	—	—	—	—	—	—	—	—	—	—	—	—	—	6	4	2	—
Carbomer 940	—	—	—	—	—	—	—	—	—	—	—	—	—	—	—	—	—	1
Triethanolamine	—	—	—	—	—	—	—	—	—	—	—	—	—	—	—	—	—	1.35
BHT	—	—	—	—	—	—	—	—	0.05	0.05	0.05	—	—	—	—	—	—	—
DI water	qs ad to 100		—	25	qs ad to 100													

HPMC: hypromellose; BHT: butylated hydroxytoluene.

**Table 3:** Physicochemical evaluation of selected topical formulations.

Formulation	Physical appearance	Color	Texture	Phase separation	Homogeneity	Immediate skin feel
C1	Opaque	Blue	Smooth	No	Homogeneous	Moisturizing, no grittiness, light, not greasy
G1	Transparent	Blue	Smooth	No	Homogeneous	Refreshing, cool, no grittiness or greasiness
G5	Transparent	Blue	Smooth	No	Homogeneous	Film formed after dry, cool, no grittiness or greasiness

**Table 4:** pH of blank and drug-loaded formulations at day 1 and at week 12.

Formulation	pH (mean ± SD)		
	Blank formulation	Drug-loaded formulations at day 1	Drug-loaded formulations at week 12
C1	3.07 ± 0.02	2.85 ± 0.03	2.90 ± 0.01
G1	6.47 ± 0.09	4.95 ± 0.06	4.96 ± 0.04
G5	6.39 ± 0.04	4.96 ± 0.04	5.05 ± 0.04

**Table 5:** Antimicrobial activity of the selected gel formulations in various dilutions against *Escherichia coli* and *Staphylococcus aureus* (*n* = 3).

Concentration of each active (%)	Zone of inhibition (mm) (mean ± SD)			
	G1		G5	
	*E. coli*	*S. aureus*	*E. coli*	*S. aureus*
0 (blank)	0	0	0	0
0 (paraben concentrate)	0	0	0	0
0.1	0	0	0	0
0.25	8.4 ± 0.2	9.1 ± 0.3	9.2 ± 0.5	9.2 ± 0.17
0.5	11.4 ± 0.1	11.9 ± 0.5	12.3 ± 0.6	11.3 ± 0.4
1	16.5 ± 0.1	18.1 ± 0.9	16.6 ± 1.3	13.9 ± 0.2
2	21.9 ± 0.6	23.1 ± 0.2	21.8 ± 0.2	17.6 ± 0.4
3	24.9 ± 0.3	26.3 ± 0.4	24.4 ± 0.4	19.7 ± 0.4
Gentamicin (control)	22.4 ± 0.15	23.5 ± 0.2	22.7 ± 1.8	21.3 ± 1.2

**Table 6:** Antimicrobial activity during the stability study.

Product	Type of storage container	Testing period	Zone of inhibition (mm) (mean ± SD)	
			Temperature	
			4°C	25°C
C1	Plastic jar	Day 1	22.7 ± 0.4	23.9 ± 0.2
		Week 3	23.4 ± 0.6	22.4 ± 0.7
		Week 6	23.1 ± 0.4	22.2 ± 0.5
		Week 9	22.8 ± 0.5	21.4 ± 0.1
		Week 12	22.1 ± 0.3	21.7 ± 0.4
	Glass jar	Day 1	23.6 ± 0.1	24.1 ± 0.8
		Week 3	21.9 ± 0.2	21.9 ± 0.2
		Week 6	22.1 ± 0.2	21.7 ± 0.5
		Week 9	22.1 ± 0.5	21.6 ± 0.3
		Week 12	21.9 ± 0.2	21.2 ± 0.2

G1	Plastic jar	Day 1	25.3 ± 0.3	24.8 ± 0.4
		Week 3	25.5 ± 0.4	25.0 ± 0.2
		Week 6	24.2 ± 0.3	24.2 ± 0.6
		Week 9	25.5 ± 0.4	25.6 ± 0.5
		Week 12	24.3 ± 0.6	25.1 ± 0.1
	Glass jar	Day 1	25.3 ± 0.3	24.7 ± 0.7
		Week 3	25.6 ± 0.3	25.1 ± 0.2
		Week 6	24.1 ± 0.3	24.2 ± 0.2
		Week 9	25.2 ± 0.3	25.7 ± 0.2
		Week 12	24.4 ± 0.2	24.7 ± 0.4

Control (gentamicin)		22.4 ± 0.8		
